# Incidence of Parental Support and Pressure on Their Children’s Motivational Processes towards Sport Practice Regarding Gender

**DOI:** 10.1371/journal.pone.0128015

**Published:** 2015-06-03

**Authors:** Diana Amado, David Sánchez-Oliva, Inmaculada González-Ponce, Juan José Pulido-González, Pedro Antonio Sánchez-Miguel

**Affiliations:** 1 Department of Physical Activity and Sport Sciences, Catholic University of Murcia (UCAM), Murcia, Spain; 2 Department of Didactic of Physical, Plastic and Musical Education, University of Cadiz (UCA), Cádiz, Spain; 3 Department of Didactic of Musical, Plastic and Corporal Expression, University of Extremadura, Cáceres, Spain; Örebro University, SWEDEN

## Abstract

Grounded in Self-Determination Theory, structural equation modeling (SEM) with the aim of examining how parental support/pressure could influence their children´s motivational processes in sport was conducted, as well as the models´ differences in operability regarding gender. The sample size was 321 children ranging in age from 10 to 16 years old who were athletes from Extremadura, and 321 parents (included only the father or mother more involved with the sport of his or her child). 175 participants were male and 146 were female from individual (n = 130), and team sports (n=191). A questionnaire was conducted to assess parental perception of support/pressure and another questionnaire was conducted to measure satisfaction of basic psychological needs, type of motivation and enjoyment/boredom showed by their children towards sport practice. Results revealed that parental pressure negatively predicted satisfaction of the basic psychological needs. It also emerged as a strong positive predictor of intrinsic motivation and negative predictor of amotivation. Moreover, intrinsic motivation emerged as positive predictor of enjoyment and a negative predictor of boredom, whereas amotivation positively predicted boredom and negatively predicted enjoyment. Furthermore, results showed there were mean differences by gender: male athletes perceived greater parental pressure. Hence, it is necessary to decrease parental pressure towards their children in sport, with the aim of making them more motivated and enjoy, promoting positive consequences.

## Introduction

Sport experiences in adolescents might give opportunities for personal growth and development in physical, cognitive, affective, social and moral domains [1, 2, 3]. So, those responsible for sport practice at this stage should attempt to promote commitment in the long term, and such commitment is related to motivational processes that determine enjoyment, positive emotional perceptions and intrinsic orientation toward an activity [4]. Nevertheless, sport experiences can also promote negative consequences such as negative emotional perceptions, anxiety, boredom and amotivation [4, 5]. Thus, youth motivation to practice sport is a key consideration to be taken into account by parents, coaches, teachers and researchers, because if we desire to involve children from an early age, it is crucial to promote a good atmosphere in those teaching ages, and it has been demonstrated that parents play an essential role in this process as principal socializing agents at this stage [6].

Parents are those responsible for the introduction of their children to a physical or sporting activity [7] and are guarantors of transport, access [8], and emotional and economic support. Sometimes they are committed to changing their familiar routines in this period [9]. All these aspects, as well as parents’ physical activity and the importance or interest they show in getting their children physically active, are the issues which determine their attitudes and positive or negative behaviour towards their children’s sport practice [10, 11, 12, 13]. Specifically, it has been demonstrated that youth athletes’ perceptions about their parents’ attitudes and behaviors in the domain of physical activity are associated with self-perception of ability, positive and negative affectivity, motivational orientation, attitudes and attraction toward physical or sport activity, as well as their behavior shown [14, 15, 16].

With this in mind, it is important to examine the involvement parents can have in their children’s sport, and the pressure they exert, because these aspects might condition children’s commitment in those activities, for both male and female participants [17, 18, 19]. Thus, parental involvement has been associated with sport participation in early ages, but there is controversy in the results found regarding gender: some authors have emphasized that this model is more important for male athletes because they follow their fathers’ model [20], whereas others works found that it is more important for girls because they tended to be less motivated for sport practice than boys and therefore need more support and encouragement [21].

Conversely, parental pressure levels are assessed during participation in physical/sport activities and are defined as the amount they push their children to compete, to get a better performance and continue practising, which was showed to be positively related with stress, and negatively associated with enjoyment and motivation [22, 23]. Respecting gender, some authors [22] conducted a study to test gender differences in athletes’ perception of their parents’ involvement or pressure, and revealed that parental pressure can promote more negative experiences in female sport practice than male practice. Therefore, continuing the study of gender differences is crucial to the enhancement of the field of knowledge about parental involvement/pressure in their children’s sport practice.

In accordance with this aspect, the quality of the relationship between parents and children is a strong predictor of stress, level of enjoyment and motivation showed by athletes [24]. From the motivation point of view, the best way that parents might become involved in their children’s sport practice would be by using some strategies to get their children intrinsically motivated through the practice of these activities. The concept of intrinsic motivation comes from Self-Determination Theory [25, 26], which studies the degree of voluntary or self-determination in people’s behavior. It develops a continuum formed from different types of motivation that vary from the more self-determined conduct or intrinsic motivation, which refers to participation in an activity because of the interest, satisfaction and pleasure of the self, to the less self-determined or amotivation, and represents an absence of motivation.

With this theory as the basis, to develop a more self-determined level of motivation, or intrinsic motivation, people have to satisfy their basic psychological needs [25] of *autonomy*, which is the sense that the individual has personal control of his or her own life; *competence*, which refers to the need to effectively carry out a behaviour in order to achieve the desired outcome, as well as the ability to handle situational demand; and *relatedness*, which refers to the need to interact, feel connected to, and be accepted by significant others. Therefore, the perceived satisfaction of these needs leads to a more self-determined or intrinsic motivation [26, 27, 28] with positive consequences for young athletes, because, as shown above, this type of motivation is related with enjoyment, curiosity, effort, the desire to participate and the intention to continue practicing an activity [29, 30].

Nevertheless, the theory also emphasises that the environment and the athletes’ significant others are fundamental elements in the satisfaction of these needs [31]. So, parental performance is very important in promoting autonomy, competence and relatedness support. Thus, an autonomy-supportive environment is characterized by the use of several strategies to involve people’s interests and preferences, encouraging them to have control of their behavior [32]. A competence-supportive environment is based on the use of strategies designed to optimize the perception of a person’s ability, giving enough time to achieve the aims, using positive feedback and recognizing effort and progress [33]. Lastly, a relatedness-supportive environment refers to the social environments in which individuals have the opportunity to develop healthy relationships with others [34]. Research has revealed that in the youth social environments, the support of these three needs determines the well-being or discomfort they show in a sporting context [35, 36].

Therefore, in accordance with the postulates Self-Determination Theory, the aim of this study was to test a structural equation model to examine the relationships between perceptions of parental support/pressure, satisfaction of the basic psychological needs, self-determination levels and the perception of consequences in their children, such as enjoyment or boredom towards physical/sport activity. Moreover, the second purpose of the work was to examine whether this model operated equally for both genders or if there were differences, understanding this concept as the characteristics that a society or culture delineates as masculine or feminine. We will try to identify these variables with the aim of optimizing and individualizing the parental involvement to positively affect their children’s sport practice in the short and long term.

According to these purposes and the literature on this topic, the first hypothesis was that parental support will positively predict satisfaction of the basic psychological needs, and parental pressure will negatively predict this satisfaction. Satisfaction of these basic psychological needs will positively predict intrinsic motivation and negatively predict amotivation. Furthermore, intrinsic motivation will positively predict enjoyment and negatively predict boredom. The second hypothesis is that the model will operate differently for both genders, as there are existing differences between them.

## Method

### Ethics Statement

As far as ethical rules are concerned, the study received the approval of the Ethics Committee of the University of Extremadura. All participants were treated in agreement with the ethical guidelines of the American Psychological Association with respect to consent, confidentiality and anonymity of the answers. Moreover, informed written consent was obtained from the parents on the behalf of the all participants involved in the study.

### Participants

The sample comprised 321 children and 321 parents (included only the father or mother more involved with the sport of his or her child). The children ranged in age from 10 to 16 years old (M = 13.44; SD = 2.92) and they were athletes from Extremadura, 175 participants were male and 146 were female from 19 different sports, both individual (n = 130), and team sports (n = 191). All athletes had a federative license, which is a license in Spain that enables an individual or a club to participate in official competitions. Sample selection was conducted by simple random sampling, and all the clusters were formed from different teams belonging to club participants in JUDEX (Extremenian Sports Games). The main objective of the JUDEX is the promotion and support of sport activities for people in school age in Extremadura. The participants are all teams and athletes belonging to Teaching Centers, Sports Clubs and Sports Associations in the Autonomous Community of Extremadura that meet the requirements of the official call, including for example being student of a school from the beginning of the course. JUDEX meets annually more than 22 different sports and more than 30,000 boys and girls in the region ranging in ages from 8 to 18, who participate in a sports program adapted to their age.

### Instruments

#### Parental perceptions of support of basic psychological needs

To assess parental perceptions of support of basic psychological needs, an adaptation of the Support of Basic Psychological Needs Questionnaire (CANPB) [37] originally created in the educative domain, was used. The introductory sentence and some other items were slightly modified to extrapolate the parental perceptions of support in sport. The scale was composed of the stem sentence «Related to the sport practice of my son/daughter…», followed by 12 items (four items per factor), which measured the support for autonomy (4 items, e.g.: “I usually ask to my son/daughter about his/her preferences regarding the sport”), support for competence (4 items e.g.: “I encourage to my son/daughter to get confidence in his/her capabilities to do well at training tasks”), and support for relatedness (4 items, e.g.: “I promote that my son/daughter have good relationships with their teammates”). This instrument showed an adequate internal consistency with Cronbach’s Alpha over .70 in all cases, revealing .73 for support for autonomy, .82 for support for competence and .88 for support for relatedness. This questionnaire only was answered by one person, the father or mother more involved with the sport of his or her child.

#### Parental perception of pressure towards their children

To assess parents’ perception of the pressure towards their children, an adaptation of the Parental Involvement Sport Questionnaire (PISQ) [38] translated into Spanish [39] was used. The adaptation consisted of a slight modification of the introductory sentence and the inclusion of some items to extrapolate parental perceptions, because the original scale was created for athletes. This scale was composed of the introductory sentence «Related to the sport practice of my son/daughter…», followed by 10 items divided into two factors. One of them referred to involvement (6 items, e.g.: “I congratulate him/her for his/her efforts after a competition”), and another factor was the pressure by parents (4 items, e.g.: “I pressure him/her to win”). This study only used parental pressure, which showed an adequate internal consistency with a value of .79. This questionnaire only was answered by one person, the father or mother more involved with the sport of his or her child.

#### Athletes’ Satisfaction of Basic Psychological Needs

To assess Athletes’ Satisfaction of Basic Psychological Needs, the Basic Psychological Needs in Exercise Scale (BPNES) [40] translated into Spanish [41] was used. This scale was composed of the introductory sentence «In sport practice…», followed by 12 items (four items per factor), which measured perception of autonomy (4 items, e.g.: “Exercises I perform are according to my interests”), competence (4 items, e.g.: “I feel that I have had a great progression regarding the final aim I have proposed”), and relatedness (4 items, e.g.: “I feel very comfortable with my teammates in training”). Internal consistency of the instrument was adequate, showing Cronbach’s Alpha scores of .71 for perception of autonomy, .71 for perception of competence, and .79 for perception of relatedness.

#### Motivation of athletes

To assess athletes’ motivation towards sport practice, a sport adaptation of the Motivation in Physical Education Questionnaire (CMEF) [42] was used. The adaptation of the instrument consisted of slightly modifying the introductory sentence and including several items to extrapolate to the context of sport. Thus, the instrument was headed by the statement «I play this sport…», followed by 20 items divided into 5 factors: intrinsic motivation, identified regulation, introjected regulation, external regulation and amotivation. In this section, two factors were selected by their opposite character, intrinsic motivation (4 items, i.e.: “Because it is fun”), and amotivation (4 items, i.e.: “I do not understand why I play this sport”). Cronbach’s Alpha coefficient showed an optimal internal consistency of both factors, revealing values of .72 for Intrinsic Motivation and .77 for Amotivation.

#### Athletes’ Enjoyment/boredom

To assess enjoyment/boredom showed by athletes toward their sport practice, an adaptation of the Enjoyment/Boredom in Sport Scale [43] translated into Spanish [44] was administered. The adaptation consisted of a slight modification of the introductory sentence and some other items to extrapolate to the context of sport. The scale started with an introductory sentence, «Respecting the sport I play…», followed by 6 items (three items for each factor) that assessed enjoyment (3 items, i.e. “I usually enjoy when I play this sport”) and boredom (3 items, i.e. “When I play this sport, I usually wish training would end quickly”). The original instrument was formed of 8 items: 4 items for enjoyment and 4 items for boredom. This study only used 6 items, because the factorial analysis showed that two items did not have adequate weights (>.30). Internal consistency of the instrument was adequate, showing values of .81 for enjoyment and .85 for boredom.

The responses of each instrument were given on a 5-point Likert scale ranging from 1 (strongly disagree) to 5 (strongly agree).

### Procedure

The study was cross-sectional; that is to say, the measurement was only taken at the middle of the season. Firstly, the main researcher contacted all the clubs belonged to JUDEX, requesting permission to include them in the study, and informing them about the goals and procedure. Later, participants were requested to answer the questions as truthfully as possible and were reassured that their responses would be strictly confidential. Informed consent was obtained from clubs and parents of athletes.

Respecting data collection, participants filled in a questionnaire answering different psychological variables included in the study in a form on internet, via Google Docs, which the participants agreed through a link provided by the researchers. Prior to this, the researchers explained to participants the meaning of the questionnaire items and they emphasized that the completion of the questionnaires was voluntary and anonymous, and they should answer honestly regarding their feelings. The questionnaires took approximately 25 minutes to complete. The principal investigator was present at all times to explain any doubts and make sure that the process was strictly followed.

### Data analysis

Initially, descriptive statistics and internal consistency estimates were calculated on all study variables. At this time, Pearson correlations were also computed to examine the relationships between the variables. All these analyses used the Statistical Package of the Social Sciences (SPSS 18.0). Next, structural equation modelling using version 18.0 of the statistical program AMOS with maximum likelihood estimation was used to address the main study purpose. In each analysis using SEM techniques, therefore, we initially evaluated the multivariate normality of the data using Mardia’s multivariate kurtosis coefficient. First, the measurement model was examined to assess the relationships between the observed indicators and their respective latent constructs.

Fit model indices were examined using the chi-square statistic: x2 value, the Comparative Fit Index (CFI), the Tuker-Lewis Index (TLI), the Incremental Fit Index (IFI), the Root Mean Square Error of Approximation (RMSEA) and the Standardized Root Mean- Square Residual (SRMR). A non-significant χ^2^ value indicates that the specified model is not significantly different from the data and thus a good fit. Values of .90 or greater for both the CFI and IFI and values of (or less) than .08 and .06 for the SRMR and RMSEA, respectively, are indicative of good model fit [45, 46].

Once the model was obtained, a factorial invariance analysis (using multi-group analysis), regarding gender, with the aim to test whether the model operates equally independently of the athletes’ gender, was conducted.

## Results

### Descriptive analysis and correlations analysis

Descriptive statistics and Pearson’s correlations of the study variables are presented in Table 1. Descriptive analysis showed that the most adaptive variables, such as support of basic psychological needs, satisfaction of these needs, intrinsic motivation and enjoyment, had greater average scores, near to the maximum of the Likert scale (5 points). Nevertheless, the most maladaptive variables such as pressure, amotivation and boredom revealed lower average values.

**Table 1:** Descriptive statistics and Pearson’s correlations among the study variables.

Variables	1	2	3	4	5	6	7	8	9	10	11
1. Parental Autonomy Support	-										
2. Parental Competence Support	.73**	-									
3. Parental Relatedness Support	.69**	.80**	-								
4. Parental Pressure	-.19**	-.17**	-.23**	-							
5. Child Autonomy Satisfaction	.13*	.09	.10	-.13*	-						
6. Child Competence Satisfaction	.16**	.08	.10	-.19**	.60**	-					
7. Child Relatedness Satisfaction	.07	.06	.13*	-.13*	.54**	.60**	-				
8. Child Intrinsic Motivation	.11	.01	.06	-.19**	.37**	.46**	.36**	-			
9. Child Amotivation	-.07	-.07	-.07	.14**	-.02	-.21**	-.21**	-.21**	-		
10. Child Enjoyment	.08	.02	.05	-.22**	.49**	.54**	.45**	.60**	-.26**	-	
11. Child Boredom	-.12*	-.12*	-.11*	.19**	-.19**	-.33**	-.30**	-.39**	.54**	-.51**	-
M	4.43	4.55	4.72	1.94	3.93	4.32	4.59	4.57	1.32	4.70	1.31
SD	.63	.63	.54	.84	.76	.64	.60	.56	.69	.61	.70
Skewness	-1.52	-1.89	-2.89	.82	-.92	-1.24	-1.91	-1.68	2.94	-2.95	3.14
Kurtosis	3.46	4.80	11.23	.02	1.30	1.90	3.75	2.55	9.40	10.20	10.74

*p < .05;

**p < .01.

Regarding correlation analysis, results showed that parental perception of support of basic psychological needs was significantly negatively related with boredom. Moreover, parental support of autonomy was significantly and positively associated with satisfaction of autonomy and competence in children; furthermore, parental support of relatedness was related with a greater perception of relatedness in children. Nevertheless, parental pressure showed a negative relationship with satisfaction of these needs, and higher association with amotivation and boredom. Finally, intrinsic motivation significantly and positively correlated with enjoyment and negatively related with boredom, whereas the relationship was inverse with amotivation.

### Structural Equation Modeling Analysis

Taking into account Self-Determination Theory, SEM was used, based on the Hierarchical Model of Motivation [47], which describes how social factors can determine the type of motivation, through satisfaction of basic psychological needs that are mediators between these social factors and motivation showed towards an activity. Furthermore, this leads to several behavioral, cognitive and affective consequences. Thus, this model included social factors (parental perception of support of the basic psychological needs and perception of support), mediators (satisfaction of the basic psychological needs of their children), types of motivation (intrinsic motivation and amotivation), and consequences (enjoyment and boredom). Previously, the measurement model was tested using the maximum likelihood method, freedom correlating the overall of latent variables. Adjusted index showed that the measurement model adequately described data: χ^2^_gl_ = 379.376_231_; CFI = .96; IFI = .96; TLI = .95; SRMR = .05 and RMSEA = .04.

Then, the SEM was conducted. Due to the Mardia multivariate coefficient’s high value (422.626), the maximum likelihood method with a bootstrapping procedure was used, which allowed us to have more robust results estimations, which were, therefore, not affected by the lack of multivariate normality [48]. The model showed the following adjusted index: χ^2^_gl_ = 461.932_244_; CFI = .94; IFI = .94; TLI = .93; SRMR = .07 and RMSEA = .05.

**Fig 1:**
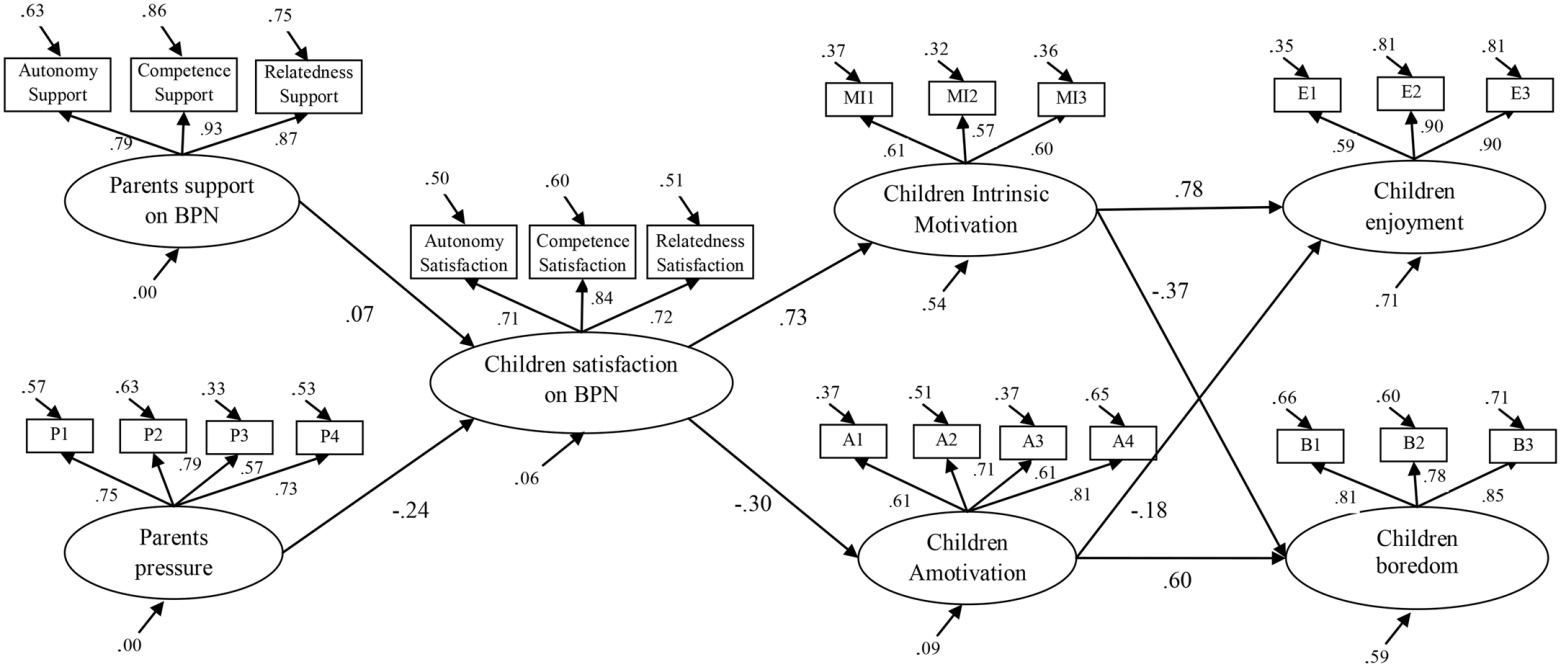
Structural equation modelling analysis. All standardized estimates *β* > ± .08 are significant (*p* < .05). *Note*. BPN = Basic Psychological Needs.

Standardized parameters are showed in Fig 1, indicating how parental pressure negatively predicted satisfaction of the basic psychological needs. Furthermore, satisfaction of the basic psychological needs was a positive strong predictor of intrinsic motivation and a negative predictor of amotivation. Finally, intrinsic motivation was a positive strong predictor of enjoyment and a negative predictor of boredom (although to a lesser extent); contrariwise, amotivation was a positive strong predictor of boredom.

The standardized indirect effects results (Table 2) revealed that pressure had a negative effect on children’s intrinsic motivation (-.18) and children’s enjoyment (-.15), and also had a positive indirect effect on children’s boredom (.11). Finally, the satisfaction of children’s basic psychological needs (BPN) had a positive indirect effect on children’s enjoyment (.63) and a negative indirect effect on children’s boredom (-.45).

**Table 2:** Indirect effect.

Variables	Effect
Parental support of BPN → Child’s intrinsic motivation	.05
Parental support of BPN → Child’s amotivation	-.02
Parental support of BPN → Child’s enjoyment	.04
Parental support of BPN → Child’s boredom	-.03
Parental pressure → Child’s intrinsic motivation	-.18*
Parental pressure → Child’s amotivation	.07
Parental pressure → Child’s enjoyment	-.15*
Parental pressure → Child’s boredom	.11*
Child’s satisfaction of BPN→ Child’s enjoyment	.63***
Child’s satisfaction of BPN→ Child’s boredom	-.45***

*p < .05;

**p < .01;

***p < .001.

### Analysis regarding gender

To test the previous model with regard to the athletes’ gender, a factorial invariance analysis, using multigroup analysis, was conducted. With the use of this technique, we aimed to assess that the model operated equally with each group. After the analysis, outcomes revealed that the four models showed statistically significant differences in chi-square, which indicated that the model created was different regarding gender.

Due to this statistically significant difference, it was decided to repeat each model for each gender, including the variables previously indicated: social factors (parental perceptions of support of the basic psychological needs and perception of pressure), mediators (satisfaction of their children’s basic psychological needs), types of motivation (intrinsic motivation and amotivation), and consequences (enjoyment and boredom). Thus, in the case of the male gender, the model showed the following adjusted index: χ^2^_gl_ = 481.710_244_; CFI = .89; IFI = .89; TLI = .88; SRMR = .09 and RMSEA = .07. Table 3 shows the standardized direct effects of the model for male athletes, where parental support of basic psychological needs had a negative effect on children’s satisfaction of these needs (-.18). Children’s satisfaction of BPN had a positive effect on their intrinsic motivation (.34) and a negative effect on their amotivation (-.33). Children’s intrinsic motivation had a positive effect on their enjoyment (.96) and a negative effect on their boredom (-.47). Finally, children’s amotivation had a negative direct effect on their enjoyment (-.11) and a positive direct effect on their boredom (.70).

**Table 3:** Comparision of standardized parameter estimates of direct effects of the structural equation modeling regarding gender.

Gender	Male gender	Female gender
Parameter	B	Β
Parental support of BPN → Child’s satisfaction of BPN	.07	.09
Parental pressure → Child’s satisfaction of BPN	-.18**	-.15
Child’s satisfaction of BPN → Child’s intrinsic motivation	.34***	.72***
Child’s satisfaction of BPN → Child’s amotivation	-.33**	-.45***
Child’s intrinsic motivation → Child’s enjoyment	.96***	.95***
Child’s intrinsic motivation → Child’s boredom	-.47**	-.64***
Child’s amotivation → Child’s enjoyment	-.11*	-.14*
Child’s amotivation → Child’s boredom	.70***	.20*

*p < .05;

**p < .01;

***p < .00.

Considering female athletes, the model showed the following adjusted index: χ^2^_gl_ = 341.033_244_; CFI = .94; IFI = .94; TLI = .93; SRMR = .07 and RMSEA = .05. Table 3 shows the standardized direct effect of the model for female athletes, where children’s satisfaction of basic psychological needs had a positive effect on their intrinsic motivation (.72) and a negative effect on their amotivation (-.45). Moreover, children’s intrinsic motivation had a positive effect on their enjoyment (.95) and a negative effect on their boredom (-.64). Finally, children’s amotivation had a negative direct effect on their enjoyment (-.14) and a positive direct effect on their boredom (.20).

The standardized indirect effects results regarding gender (Table 4) revealed that pressure had a negative effect on children’s intrinsic motivation (-.17 male and-.14 female) and enjoyment (-.13 male and-.14 female) in both genders. Moreover, the satisfaction of children’s basic psychological needs (BPN) had a positive indirect effect on children’s enjoyment (.47 male and .69 female), and a negative indirect effect on children’s boredom (-.34 male and-.57 female) in both genders.

**Table 4:** Comparision of standardized parameter estimates of indirect effects of the structural equation modeling regarding gender.

Gender	Male gender	Female gender
Parameter	B	β
Parental support of BPN → Child’s intrinsic motivation	.05	.04
Parental support of BPN → Child’s amotivation	-.02	-.02
Parental support of BPN → Child’s enjoyment	.04	.04
Parental support of BPN → Child’s boredom	-.03	-.03
Parental pressure → Child’s intrinsic motivation	-.17*	-.14*
Parental pressure → Child’s amotivation	.07	.08
Parental pressure → Child’s enjoyment	-.13*	-.14*
Parental pressure → Child’s boredom	.09	.12
Child’s satisfaction of BPN→ Child’s enjoyment	.47***	.69***
Child’s satisfaction of BPN→ Child’s boredom	-.34**	-.57***

*p < .05;

**p < .01;

***p < .00.

## Discussion

In accordance with the aims of the study, the first hypothesis was that parental support positively predicted the satisfaction of their children’s basic psychological needs, and contrariwise, parental pressure negatively predicted the satisfaction of their children’s basic psychological needs. Satisfaction of these needs would positively predict intrinsic motivation and negatively predict amotivation. Moreover, intrinsic motivation would positively predict enjoyment and negatively predict boredom. In this regard, results showed that parental pressure negatively predicted satisfaction of basic psychological needs, and these needs emerged as a strong positive predictor of intrinsic motivation and a negative predictor of amotivation. Lastly, intrinsic motivation was a positive strong predictor of enjoyment and a negative predictor of boredom (to a lesser extent), whereas amotivation emerged as a strong predictor of boredom.

Therefore, it is important to avoid the idea that parental pressure on their children to win, to improve continually, to make more of an effort, to achieve their aims, because it has been shown that pressure negatively predicted satisfaction of basic psychological needs. Moreover, indirect effects emphasized that parental pressure also negatively predicted intrinsic motivation and enjoyment [22], and positively predicted boredom. This highlighted the negative aspects of parental pressure on their children towards sport practice, because it indicated that the more constraints athletes felt, the lesser the perception of competence, autonomy and relatedness they will feel. This issue will lead to less enjoyment of practice, amotivation and boredom, which, in the long run, will be translated into a lack of commitment, concluding with dropout from an activity, as was previously indicated by many authors [49, 50, 29, 30].

Continuing with the analysis of the results, satisfaction of the basic psychological needs emerged as a positive predictor of intrinsic motivation and enjoyment [26, 27, 28]. Furthermore, satisfaction of these needs did not only predict positive consequences but emerged as a negative strong predictor of boredom. Lastly, as athletes have a greater perception of their competence, autonomy and relatedness, they will get more enjoyment from the practice itself, moving away from amotivation and boredom.

Finally, the model analyzed the relationship between types of motivation and different outcomes, showing how intrinsic motivation positively predicted enjoyment and negatively predicted boredom. These results are logical, because postulates of SDT [51] indicate that motives of practice within intrinsic motivation are related with satisfaction, pleasure, happiness and fun, and obviously, with enjoyment. Thus, it is logical that those athletes who develop these feelings get more enjoyment from sport practice, and feel less boredom. Previous research already found intrinsic motivation to be a positive predictor of enjoyment [30]. Furthermore, results also revealed that amotivation positively predicted boredom and negatively predicted enjoyment; that is to say a lower self-determination level of motivation is related with less adaptive consequences [51].

Therefore, according to the results found in contrast with the first hypothesis, it is shown that this hypothesis is partially confirmed because parental support of the basic psychological needs did not emerge as a positive predictor of satisfaction of these needs in athletes. These outcomes might be explained thus: perhaps parents play an important role in reducing the pressure on their children, but the crucial issue of supporting basic psychological needs comes from coaches, as was emphasized by previous studies [52, 53, 54].

Regarding the second hypothesis, which indicated that the model designed to predict athletes’ motivational processes according to their parents’ behaviors operated differently for each gender, there were differences between them. Thus, data revealed that this hypothesis might be confirmed, because there are differences between both genders in the model indicated. Therefore, the model was not invariant regarding gender, so it is an important contribution to the scientific community. In this regard, results showed that the principal difference between male and female was that male’s perceived parental pressure negatively predicted satisfaction of basic psychological needs. Nevertheless, regarding female participants, neither parental support nor pressure emerged as predictors of these needs in sport practice. These results are consistent with some authors [20], who emphasized the importance of fathers as models for their sons to follow in sport practice. Due to this aspect, perhaps, they give more importance to pressure received from their fathers, and this issue leads to a decrease in the satisfaction of their basic psychological needs, with negative consequences associated with continuity in the sport; according to the analysis of the indirect effects of the model, pressure would also decrease intrinsic motivation and enjoyment.

Therefore, the main conclusions of the study emphasize the importance of basic psychological needs to achieve an intrinsic motivation in the context of sport, which leads to the appearance of adaptive consequences, such as enjoyment in both genders. Moreover it is important to avoid parental pressure, because it is related with a lower satisfaction of basic psychological needs, lower intrinsic motivation and less enjoyment. Thus, it is crucial to develop teaching programs with the aim of teaching parents certain strategies to avoid pressure and promote support of their children’s sport, making them aware of the relevance of appropriate behavior towards their children’s sport. In this regard, some strategies are: parental education about how to be appropriately involved in their children’s sport practice, and how they can be supportive to get them more motivated and satisfied with their activity; learning to be aware of their own acts (the number of times they encourage their children and how they do it, how they can help their children with transport, nutrition, healthy habits, etc).

Nevertheless, although the current work reveals very interesting results about the role of parents in their children’s motivational processes towards sport regarding gender, it is necessary to take into account further research to resolve certain limitations found in this study. According to these limitations, it is important to note that this research did not measure real parental behaviors but the children’s perceptions of parental behavior. The difference in participants’ ages might point to different interests or a different perception of pressures. Moreover, this work did not assess aspects such as academic training or parents’ social status, and further, the instruments used are adaptations of the physical education domain. However, in future investigations, apart from attempting to alleviate these limitations, it might be interesting to replicate the study comparing the influence of fathers and mothers separately, because it is usually one of them that is most heavily involved in his/her children’s practice. Also, including their level of education and economical income as socio-metric variables could give us more of an insight into reality. Another thing to consider could be an analysis of the differences between the variables studied and gender regarding the type of sport, examining gender stereotypes in depth. They are still present in sport practice, mainly in school age.
